# Combined Local Anesthetic Infiltration Versus Intrathecal Morphine for Postcesarean Analgesia: A Pilot Randomized Double-Blind Trial

**DOI:** 10.5152/eurasianjmed.2026.261445

**Published:** 2026-09-21

**Authors:** Ramazan İnce, Mehmet Akif Yılmaz, Miraç Selcen Özkal Yalın, Ayşenur Dostbil, Kamber Kaşali, Gamze Nur Cimilli Şenocak, Mehmet Aksoy, Selvihan Tapanoğlu Karaca, Mehmet Sercan Orbak, İlker İnce, Agah Abdullah Kahramanlar

**Affiliations:** 1 Department of Anesthesiology and Reanimation, Erzurum Regional Training and Research Hospital, Health Sciences University, Erzurum, Türkiye; 2 Department of Anesthesiology and Reanimation, Ataturk University School of Medicine, Erzurum, Türkiye; 3 Anesthesiology Clinical Research Ofﬁce, Ataturk University, Erzurum, Türkiye; 4 Department of Biostatistics, Ataturk University School of Medicine, Erzurum, Türkiye; 5 Department of Obstetrics and Gynecology, Ataturk University School of Medicine, Erzurum, Türkiye; 6 Department of Obstetrics and Gynecology, Erzurum Regional Training and Research Hospital, Health Sciences University, Erzurum, Türkiye; 7 Milton S. Hershey Medical Center, Penn State University, PA, USA

**Keywords:** Intraperitoneal local anesthetic, intrathecal morphine, postcesarean analgesia, rectus aponeurosis infiltration, wound infiltration

## Abstract

**Background:**

This exploratory pilot randomized double-blind trial compared postoperative analgesic outcomes of combined local wound infiltration, rectus aponeurosis infiltration, and intraperitoneal local anesthetic instillation (LWI+RAI+IPLA) with intrathecal morphine (ITM) after cesarean delivery.

**Methods:**

Forty-six women undergoing elective cesarean delivery under spinal anesthesia were randomized to receive either ITM (150 µg) or LWI+RAI+IPLA using a bupivacaine–lidocaine–epinephrine mixture. Both groups received standardized spinal bupivacaine–fentanyl and postoperative multimodal analgesia. The primary endpoint was 24-hour rescue morphine consumption. Secondary endpoints included pain scores, time to first rescue opioid request, and opioid-related adverse effects.

**Results:**

Rescue morphine consumption was numerically higher with LWI+RAI+IPLA than with ITM, but the difference was not significant (1.3 ± 2.22 vs 0.34 ± 0.98 mg; *P* = .075). Pain scores at rest and during movement did not differ significantly at any time point. Time to first rescue opioid request was longer with ITM (23.04 hours [95% CI, 21.30-24.79] vs 18.70 hours [95% CI, 15.36-22.04]; log-rank *P* = .032). Nausea/vomiting was more frequent with ITM but was not significant (30.4% vs 8.7%; *P* = .135), whereas pruritus was significantly more frequent with ITM (34.8% vs 8.7%; *P* = .032). No respiratory depression occurred.

**Conclusion:**

Twenty-four-hour opioid consumption and pain scores did not differ significantly between groups. Intrathecal morphine prolonged the time to first rescue opioid request, whereas LWI+RAI+IPLA caused less pruritus. Because this pilot study was not powered for equivalence or noninferiority and showed a floor effect in opioid consumption, larger adequately powered trials are needed.

**Main Points**

- In this exploratory pilot randomized trial, 24-hour rescue morphine consumption and pain scores did not differ significantly between local wound infiltration, rectus aponeurosis infiltration, and intraperitoneal local anesthetic instillation (LWI+RAI+IPLA) and 150 µg intrathecal morphine (ITM); however, these findings should not be interpreted as evidence of comparability.
- Intrathecal administration of morphine prolonged the time to first rescue opioid requests, which is consistent with its long-acting neuraxial profile.
- Pruritus occurred significantly more frequently with ITM, whereas the multimodal local anesthetic approach was associated with fewer opioid-related adverse effects.
- The LWI+RAI+IPLA technique may represent a practical nonneuraxial, local anesthetic-based alternative when neuraxial opioids are contraindicated or undesirable, but larger, adequately powered trials are needed to define its comparative effectiveness.

## Introduction

Cesarean delivery (CD) is the most commonly performed major obstetric surgery worldwide, and global rates continue to increase because of evolving obstetric practices and maternal preferences.^1^ Effective postoperative pain control is essential for optimizing maternal recovery, as inadequate analgesia negatively affects early mobilization, breastfeeding success, maternal–infant bonding, and overall postoperative well-being.^2^^,^^3^ Moreover, poorly controlled acute postcesarean pain is strongly associated with the later development of persistent postoperative pain and postpartum depression.^4^^,^^5^

Current guidelines recommend a multimodal analgesic approach that combines systemic and regional techniques to target both somatic pain from the abdominal incision and visceral pain originating from uterine manipulation and peritoneal irritation.^6^ Intrathecal morphine (ITM) remains the gold standard for postcesarean analgesia because of its prolonged and reliable opioid-sparing effect.^6^^,^^7^ Doses of 100-200 μg provide effective analgesia but may be accompanied by pruritus, nausea, vomiting, and dose-dependent respiratory depression, which can decrease maternal satisfaction and delay recovery.^8^^,^^9^ Consequently, alternative or adjunctive opioid-sparing techniques are being increasingly explored, particularly for women in whom neuraxial opioids are contraindicated.

Local anesthetic-based approaches, such as intraperitoneal local anesthetic instillation (IPLA), local wound infiltration (LWI), and rectus sheath or fascial plane infiltration, target peripheral nociceptive pathways (Figure 1). These techniques aim to reduce afferent visceral signaling, modulate inflammatory responses, and provide site-specific analgesia without the systemic side effects associated with opioids.^10^^,^^11^ Evidence suggests that IPLA may reduce early postoperative visceral pain and early opioid requirements, whereas LWI decreases incisional pain intensity and postoperative opioid use.^12^^-^^14^ When combined, these techniques may offer additive or synergistic benefits by addressing both peritoneal and abdominal wall pain generators. However, the literature remains limited regarding the combined use of the IPLA, LWI, and rectus aponeurosis infiltration (RAI), and no prior study has directly compared this multimodal local anesthetic strategy with intrathecal morphine during cesarean delivery under spinal anesthesia. Determining how this opioid-sparing combination performs relative to ITM is clinically relevant, especially for settings where neuraxial opioids are unavailable or contraindicated.

Therefore, this exploratory, pilot randomized double-blind trial was designed to compare postoperative analgesic outcomes between LWI+RAI+IPLA and ITM in women who underwent elective cesarean delivery under spinal anesthesia. The primary hypothesis was that 24-hour rescue morphine consumption would not differ significantly between groups. Secondary outcomes included visual analog scale (VAS) pain scores at rest and during movement, time to first rescue from opioid requests, and opioid-related adverse effects.

## Material and Methods

This exploratory pilot, prospective, randomized, double-blind clinical trial was conducted at a tertiary university hospital between June 16, 2022 and November 11, 2023. Ethical approval was obtained from the Ethics Committee of Atatürk University Clinical Research on March 31, 2022 (B.30.2.ATA.0.01.00/339), and the study was registered at ClinicalTrials.gov (NCT05405049). The prespecified primary outcome was total opioid consumption during the first 24 postoperative hours; in the present study protocol, the only postoperative opioid used for rescue analgesia was subcutaneous morphine; therefore, “total opioid consumption” corresponds to the cumulative rescue morphine dose over 0-24 hours. The prespecified secondary outcome was pain intensity assessed via a 0-10 VAS at 2, 4, 6, 12, and 24 hours post-operatively (at rest and during movement). The outcomes reported in this manuscript are aligned with the trial registration record, and no outcome changes were made after trial commencement. All participants provided written informed consent, and the study was performed in accordance with the Declaration of Helsinki and national research regulations. This study did not use AI and/or LLM tools during the preparation of this manuscript.

A total of 46 pregnant women aged 18-50 years, who were classified as ASA II and who had full-term singleton pregnancies and who were scheduled for elective cesarean delivery under spinal anesthesia, were enrolled. Patients were excluded if they had contraindications to neuraxial anesthesia, a BMI > 35 kg/m^2^, an ASA score ≥ III, diabetes, preeclampsia, cardiovascular disease, chronic or neuropathic pain, previous abdominal surgery, intraoperative opioid administration, conversion to general anesthesia, excessive bleeding or uterine atony, surgical drains near the infiltration area, a cognitive inability to understand the VAS, or a history of drug abuse or psychiatric illness.

Randomization was performed via a computer-generated table prepared by an independent statistician. Allocation concealment was ensured via sequentially numbered, opaque, sealed envelopes, which were opened immediately before spinal anesthesia. The study medications were prepared in identical syringes to ensure blinding. Patients, surgeons, anesthesia providers, and postoperative evaluators remained blinded to group allocation throughout the study. Because certain exclusion criteria could only become apparent intraoperatively after randomization, the study protocol prospectively specified that participants excluded for such reasons would be replaced through subsequent randomization until a prespecified analyzable sample size of 23 participants per group was achieved. These intraoperative criteria included surgical complications, deviations from the planned surgical procedure, and drain placement in the infiltration area. Replacement participants underwent the same eligibility assessment, randomization, allocation concealment, and blinding procedures as the initially enrolled participants did.

Standard ASA monitoring was applied upon arrival at the operating room. Spinal anesthesia was administered at the L3-L4 or L4-L5 interspace via 0.5% hyperbaric bupivacaine and 15 μg fentanyl. The ITM group received an additional 150 μg of ITM, whereas the LWI+RAI+IPLA group received the same spinal mixture without morphine. The 150 µg ITM dose was selected a priori on the basis of dose-finding evidence and contemporary guidance. In a dose–response ED90 study in postcesarean patients, the ED90 for intrathecal morphine was reported to be 150 µg, suggesting that this dose is effective.^15^ While procedure-specific recommendations such as PROSPECT often favor lower doses (50-100 µg) to minimize side effects, other evidence-based guidance supports 100-150 µg as an appropriate low-dose range; therefore, 150 µg was chosen to ensure reliable 24-hour analgesia within the multimodal protocol. Hemodynamic variables were managed according to institutional protocols. All patients received prophylactic ondansetron and oxytocin after delivery.

Following delivery and uterine closure, patients in the LWI+RAI+IPLA group received a total of 30 mL of local anesthetic solution containing 15 mL of 0.5% bupivacaine, 15 mL of 2% lidocaine, and epinephrine (1 : 200 000). From this solution, 10 mL was instilled intraperitoneally over the uterine peritoneum, 10 mL was infiltrated into the rectus aponeurosis, and 10 mL was infiltrated subcutaneously at the wound site. The morphine group received 30 mL of isotonic saline in identical volumes and locations to preserve blinding. All intraperitoneal instillations and abdominal wall infiltrations (LWI+RAI+IPLA or saline placebo) were performed by the obstetric surgeon performing the cesarean delivery, following a standardized protocol; the surgeon remained blinded to group allocation, as syringes were prepared by an independent staff member.

### Intervention and Dose Selection

The local anesthetic regimen was designed to provide rapid onset (lidocaine) together with a longer duration of analgesia (bupivacaine), whereas epinephrine (1 : 200 000) was added to prolong the local effect and reduce systemic uptake.^16^ Bupivacaine, like lidocaine, is an amide local anesthetic but provides a longer duration of action.^16^ Moreover, pharmacological evidence indicates that epinephrine delays local anesthetic absorption, thereby prolonging the effect and reducing the risk of systemic toxicity.^16^ Intraperitoneal local anesthetic instillation has previously been shown to improve early postcesarean analgesia, and a randomized trial of cesarean delivery reported the use of intraperitoneal instillation with a bupivacaine–lidocaine combination.^13^^,17^ With respect to systemic exposure, in women undergoing total abdominal hysterectomy, intraperitoneal lidocaine resulted in low systemic exposure, with a mean peak serum concentration of 0.4 µg/mL and the highest observed concentration of 0.87 µg/mL.^18^ Local wound/fascial infiltration is supported by prior evidence, including systematic review data.^12^ The total administered doses were 75 mg of bupivacaine and 300 mg of lidocaine (plus 150 µg of epinephrine), which are within the commonly cited maximum recommended single-dose limits for healthy adults; when local anesthetics are combined, doses are kept well below individual maxima to minimize additive toxicity risk.^19^ On the basis of the lowest recorded body weight in this cohort, the administered doses corresponded to conservative mg/kg values, remaining below commonly cited maximum limits when epinephrine was used.

Postoperatively, all patients received standardized multimodal analgesia consisting of intravenous dexketoprofen (50 mg every 12 hours) and intravenous paracetamol (15 mg/kg every 6 hours) during the first 24 hours. Rescue analgesia consisted of subcutaneous morphine (2 mg boluses, maximum 20 mg within 4 hours) when the VAS pain score exceeded 3. Nausea was treated with intravenous ondansetron, and pruritus was treated with oral cetirizine. Respiratory rate and sedation were monitored using the Pasero Opioid-Induced Sedation Scale for 24 hours.

### Safety Monitoring

In addition to standard ASA monitoring, patients were observed clinically for potential local anesthetic systemic toxicity (LAST) symptoms (e.g., perioral numbness/paresthesia, tinnitus, metallic taste, agitation, and seizures) via continuous electrocardiogram (ECG) and serial blood pressure/heart rate monitoring in the operating room during the early recovery period. A 20% lipid emulsion “LAST rescue” kit was immediately available, and institutional management followed published American Society of Regional Anesthesia and Pain Medicine (ASRA) guidelines.^20^^-^^23^ No clinical features consistent with LAST occurred.

Pain scores at rest and during movement were assessed via a 0-10 VAS at 2, 4, 6, 12, and 24 hours postoperatively. Other collected variables included age, anthropometric measures, gestational age, operative time, sensory block level, time to first opioid request, time to return of bowel function, conversion to general anesthesia, and maternal satisfaction. The primary outcome was total rescue morphine consumption within the first 24 postoperative hours, excluding intrathecal morphine administered during spinal anesthesia. The secondary outcomes were the VAS score; time to first rescue analgesia, and opioid-related adverse effects, such as nausea, vomiting, pruritus, sedation, and respiratory depression.

### Sample Size

In accordance with the methods of Anthony A. Bamigboye et al^24^, 400 ± 147 mg of pethidine was taken during the first 24 hours after surgery from the group that received an intraperitoneal drip in addition to local anesthetic infiltration into the wound. The G*Power program (Heinrich Heine University Düsseldorf, Düsseldorf, Germany) was used to determine that, with 80% power and a 95% CI, 23 participants per group (a total of 46 participants) would be needed for the study in order for the difference in consumption between the 80 mg group and the morphine group to be statistically significant. This effect size was selected because the data from Bamigboye et al represented the most comparable available evidence at the time of protocol development for a combined intraperitoneal and wound infiltration approach after cesarean delivery. However, the present trial used a contemporary multimodal analgesic regimen and low-dose subcutaneous morphine rescue analgesia rather than pethidine. Therefore, the assumed effect size may have overestimated the expected opioid requirement under the present study conditions.

### Statistical Analyses

A prespecified statistical analysis plan (SAP) was finalized prior to database lockdown and unblinding. The primary analysis compared cumulative rescue morphine consumption over 0-24 hours between groups using a 2-sided significance level of 0.05; secondary outcomes were analyzed as supportive/exploratory. The analyses were conducted in the per-protocol population, defined as randomized participants who completed the assigned intervention without meeting a prespecified intraoperative exclusion criterion. Statistical analyses were performed using IBM Statistical Package for the Social Sciences Statistics for Windows, version 20.0 (IBM Corp., Armonk, NY, USA). Continuous variables were assessed for a normal distribution via graphical methods and the Shapiro–Wilk test. Normally distributed variables were compared via the independent samples *t-*test, whereas nonnormally distributed variables were analyzed via the Mann–Whitney *U-*test. Repeated measures of pain scores were evaluated via repeated-measures analysis of variance (ANOVA) for normally distributed data and the Friedman test for nonparametric data. When applicable, post hoc analyses were conducted via standard appropriate corrections. The time to first rescue opioid requirement was analyzed using the Kaplan–Meier method. Patients who did not require rescue opioid analgesia during the 24-hour follow-up period were censored at 24 hours. The survival curves were compared using the log-rank (Mantel–Cox) test.

Categorical variables were analyzed using the Pearson chi-square test or Fisher’s exact test according to expected cell frequencies. Postoperative adverse events were compared between groups using Fisher’s exact test. Risk differences and risk ratios were calculated with their corresponding 95% CI. Risk differences were calculated as the incidence in the ITM group minus that in the LWI+RAI+IPLA group, and risk ratios were calculated using the LWI+RAI+IPLA group as the reference group. Continuous variables are presented as the mean ± SD with 95% CI and median (minimum-maximum), whereas categorical variables are presented as numbers and percentages, as appropriate. Between-group standardized effect sizes for continuous variables were reported as Cohen’s *d* and calculated using the pooled standard deviation. A 2-tailed *P* value of < .05 was considered to indicate statistical significance. The authors declared that no Artificial Intelligence tool was used in the preparation of the manuscript

## Results

A total of 115 women were screened for eligibility. Initially, 46 eligible participants were randomized. During surgery, 8 participants met a prespecified intraoperative exclusion criterion because of drain placement in the infiltration area and were therefore excluded after randomization (LWI+RAI+IPLA group, n = 5; ITM group, n = 3). In accordance with the prospectively specified replacement procedure, 8 additional eligible participants were subsequently randomized using the same allocation and blinding procedures. Thus, a total of 54 participants were randomized throughout the study, and 46 participants completed the study and were included in the per-protocol analysis, with 23 participants in each group (Figure 2).

The baseline demographic characteristics and duration of surgery were comparable between the groups, except for height, which was significantly greater in the LWI+RAI+IPLA group [164.43 ± 5.54 cm (95% CI: 161.90-166.92) vs 160.56 ± 5.55 cm (95% CI: 158.16-162.97); *P* = .023] (Table 1).

The prespecified primary endpoint was cumulative rescue morphine consumption during the first 24 postoperative hours. Postoperative outcomes are summarized in Table 2. The time to first postoperative rescue opioid requirement was evaluated using Kaplan–Meier survival analysis, and the survival curves were compared using the log-rank (Mantel–Cox) test (Figure 3). In the ITM group (n = 23), 2 patients (8.7%) required rescue opioid analgesia during the 24-hour follow-up period, whereas 21 patients (91.3%) were censored. In the LWI+RAI+IPLA group (n = 23), 8 patients (34.8%) required rescue opioid analgesia, whereas 15 patients (65.2%) were censored. The Kaplan–Meier-estimated restricted mean time to first rescue opioid requirement was 23.04 hours (SE, 0.89; 95% CI: 21.30-24.79) in the ITM group and 18.70 hours (SE, 1.70; 95% CI: 15.36-22.04) in the LWI+RAI+IPLA group. The log-rank test demonstrated a statistically significant difference between the groups (*χ*^2^ = 4.58, df =1, *P* = .032), indicating that patients in the intrathecal morphine group remained free from rescue opioid requirements for a significantly longer period. Total rescue morphine consumption during the first 24 hours was numerically greater in the LWI+RAI+IPLA group than in the ITM group, but this difference was not statistically significant (1.3 ± 2.22 mg [95% CI: 0.17-1.83] vs 0.34 ± 0.98 mg [95% CI: −0.24 to 1.10]; *P* = .075). The time to first ambulation, time to first bowel movement, and patient satisfaction did not significantly differ between the groups (Table 2). Notably, the rescue of morphine consumption resulted in a marked floor effect. As shown in Table 2, the median 0-24 hours rescue morphine dose was 0 mg in both groups (0 [0-4] in the ITM group and 0 [0-8] in the LWI+RAI+IPLA group), indicating that most participants did not require rescue opioids during the 24-hour follow-up. Accordingly, the low mean values reflect rescue morphine use in a limited number of participants rather than a uniform opioid requirement across the cohort.

Postoperative VAS scores at rest and during movement are summarized in Table 3. Between-group comparisons revealed no statistically significant differences at any postoperative time point, either at rest or during movement. At rest, VAS scores tended to be numerically higher in the LWI+RAI+IPLA group at 4, 6, and 12 hours, whereas 24-hour scores were identical between groups. During movement, VAS scores were numerically greater in the LWI+RAI+IPLA group at 4, 6, and 12 hours, but these differences were not statistically significant. Within-group analysis revealed no significant temporal change in the ITM group, whereas the LWI+RAI+IPLA group demonstrated significant changes over time both at rest (*P* = .033) and during movement (*P* = .004), with post hoc differences observed between 2 hours and 6 hours at rest and between 2 hours and 6 hours and between 2 hours and 12 hours during movement.

The distributions of the maximal sensory block levels (T1-T6) were similar between the groups, with no significant intergroup differences. Postoperative adverse events were compared between groups using Fisher’s exact test. The incidence of nausea/vomiting was 30.4% (n = 7) in the ITM group and 8.7% (n = 2) in the LWI+RAI+IPLA group, and the between-group difference was not statistically significant (risk difference, 21.7 percentage points [95% CI: −0.3 to 43.8]; risk ratio, 3.50 [95% CI: 0.81-15.09]; *P* = .135). The incidence of pruritus was 34.8% (n = 8) in the ITM group and 8.7% (n = 2) in the LWI+RAI+IPLA group, and the between-group difference was statistically significant (risk difference, 26.1 percentage points [95% CI: 3.5-48.7]; risk ratio, 4.00 [95% CI: 0.95-16.84]; *P* = .032). No episodes of respiratory depression were observed in either group.

Overall, postoperative analgesic outcomes were similar between the groups across the measured time points; however, pruritus occurred significantly less frequently in the LWI+RAI+IPLA group.

## Discussion

Importantly, the primary inference of the trial was based on the prespecified 0-24 hours cumulative rescue morphine outcome, whereas pain score trajectories were evaluated as secondary outcomes. In this randomized, double-blind study, no statistically significant difference was observed between LWI+RAI+IPLA and ITM at 0-24 hours of rescue morphine consumption or VAS pain scores during the first 24 hours after cesarean delivery. The time to first rescue opioid request was longer in the ITM group, which is consistent with the prolonged duration of hydrophilic neuraxial opioids. The very low cumulative rescue morphine requirement, with a median value of 0 mg in both groups, suggests a floor effect under the standardized multimodal analgesia regimen, which may have limited the ability of 0-24 hours rescue opioid consumption to discriminate between groups. Therefore, the findings should be interpreted in the context of the totality of outcomes, including pain scores over time, time to first rescue opioid request, and opioid-related adverse effects. Importantly, statistical nonsignificance should not be equated with the absence of clinical relevance or with evidence of equivalence. The observed numerical differences, together with the pilot sample size and low rescue opioid requirement, should be interpreted cautiously and considered exploratory. This discrepancy between the opioid consumption assumed during sample size estimation and the very low rescue morphine requirement observed in the present study should be considered when interpreting the primary outcome. Although the assumed effect size was clinically reasonable on the basis of the evidence available during protocol development, the contemporary multimodal analgesic regimen used in this trial likely reduced overall opioid requirements and limited the discriminatory ability of cumulative 24-hour rescue morphine consumption.^6^

Previous studies evaluating intraperitoneal lidocaine have reported no significant differences in total opioid use or pain scores during the first 24 hours compared with placebo, even when movement-related pain appeared slightly lower in the early postoperative period.^17^ Similarly, the literature on wound infiltration demonstrates that this technique may reduce early postoperative pain and opioid use, although the analgesic benefit becomes more apparent in patients who do not receive ITM.^12^ These findings support the concept that peripheral local anesthetic techniques have limited incremental value when neuraxial morphine is already part of the analgesic regimen.

Kainu et al^25^ compared continuous wound infiltration with ITM and reported lower opioid consumption in the ITM group, whereas pain scores were similar between groups. These results mirror those of the current study, in which pain scores were similar between groups and rescue opioid consumption did not differ significantly.

In contrast, Bamigboye et al^24^ investigated LWI+RAI+IPLA under general anesthesia and reported a significant reduction in early postoperative opioid use and lower pain scores during the first hours after cesarean delivery. Although the present study did not demonstrate statistically significant between-group differences in early postoperative VAS scores, the findings of Bamigboye et al support the rationale that targeting both peritoneal and abdominal wall nociception may enhance postoperative analgesia when ITM is not used.

The significant within-group temporal changes in VAS scores in the LWI+RAI+IPLA group, observed both at rest and during movement, may reflect the gradual waning of local anesthetic effects over the first postoperative day. In contrast, the absence of significant temporal change in the ITM group is consistent with the prolonged analgesic profile of neuraxial morphine.^26^ The addition of epinephrine to the local anesthetic mixture may have extended tissue retention and contributed to sustained analgesic effects, a benefit supported by previous research.^27^

The greater incidence of pruritus in the ITM group is consistent with the known dose-related side effect profile of neuraxial morphine. Although some procedure-specific recommendations favor lower ITM doses to minimize side effects, 150 µg remains within the evidence-supported low-dose range used in randomized comparisons and guidance documents and is also consistent with dose-finding data reporting an ED90 of 150 µg for postcesarean analgesia.^6^^,^^15^^,28^ In this cohort, nausea/vomiting occurred more frequently in the ITM group than in the LWI+RAI+IPLA group, although the difference was not statistically significant. In contrast, the incidence of pruritus was significantly greater in the ITM group, which is consistent with the known opioid-related adverse effect profile of intrathecal morphine and with findings from previous trials.^25^^,^^26^ Therefore, the higher pruritus rate observed in the ITM group may partly reflect the selected 150 µg dose, and future studies comparing LWI+RAI+IPLA with lower ITM doses may better define the balance between analgesic efficacy and opioid-related adverse effects.

Taken together, these results suggest that LWI+RAI+IPLA may represent a practical nonneuraxial local anesthetic-based alternative in settings where neuraxial opioids are undesirable or contraindicated. However, because rescue morphine consumption was numerically higher and the time to first opioid requirement was shorter in the LWI+RAI+IPLA group, this technique should not be interpreted as equivalent or superior to ITM on the basis of the present pilot data. Because the technique is simple, inexpensive, and does not require ultrasound guidance or specialized training, it may be considered in patients who cannot receive neuraxial opioids or in settings with limited resources, while acknowledging that definitive comparative effectiveness requires larger adequately powered studies.^13^

Epinephrine (1 : 200 000) was included to prolong the duration of local infiltration analgesia and reduce systemic absorption, which is consistent with prior pharmacologic and clinical observations. Importantly, intraperitoneal and abdominal wall administration was performed after delivery and uterine closure, thereby minimizing any potential fetal exposure. Although no clinical findings suggestive of local anesthetic systemic toxicity were observed under continuous perioperative monitoring, plasma concentrations of lidocaine and bupivacaine were not measured. Therefore, systemic safety interpretations should be limited to the absence of clinically detected toxicity rather than definitive pharmacokinetic safety confirmation. Future studies incorporating serial plasma local anesthetic measurements could provide a more comprehensive assessment of systemic exposure and safety following combined intraperitoneal and abdominal wall administration.^29^

### Clinical Implications

When neuraxial opioids are contraindicated, unavailable, or intentionally avoided, a multimodal local anesthetic strategy combining intraperitoneal instillation, rectus aponeurosis infiltration, and wound infiltration may be considered part of a nonneuraxial multimodal postcesarean analgesic regimen. This approach is simple and low cost, does not require ultrasound guidance or specialized equipment, and can be integrated with standard nonopioid multimodal analgesia. Nevertheless, the present pilot data do not establish equivalence or noninferiority to ITM, and clinicians should recognize that the time to first opioid requirement was longer with ITM. Dose limits should be respected, vigilance for local anesthetic systemic toxicity should be maintained, and definitive comparative effectiveness requires larger adequately powered trials with prespecified margins and patient-centered recovery outcomes.

### Limitations

This study has several limitations. First, postoperative sensory block assessments were not conducted; therefore, block regression (spinal “washout”) was not formally evaluated via a standardized sensory/motor scale, and early postoperative pain scores and rescue opioid requirements may have been influenced by residual neuraxial anesthesia. This may have particularly affected early postoperative VAS scores, during which time residual spinal anesthesia could have reduced the apparent pain burden in both groups. Although randomization should balance this between groups, future studies should incorporate predefined block regression criteria and report interval-based rescue dosing to improve endpoint sensitivity. Because 8 participants were excluded after randomization and were replaced in accordance with the prospectively specified protocol, the analysis represents a per-protocol rather than a strict intention-to-treat approach. Although this replacement strategy was designed to preserve the target analyzable sample size, postrandomization exclusions and participant replacement may have introduced attrition and/or replacement bias. This potential source of bias should be considered when interpreting the internal validity and generalizability of the findings. Second, rescue opioid use was summarized as cumulative 0-24 hours morphine consumption, and detailed time-stamped rescue dosing information (e.g., number of 2-mg boluses and their distribution across postoperative hours) was not available for analysis, limiting temporal characterization of analgesic patterns. Third, because follow-up was limited to the first 24 postoperative hours, longer-term maternal recovery outcomes, breastfeeding-related outcomes, quality of mobilization, functional recovery, and persistent postoperative pain could not be evaluated. Fourth, although the sample size was calculated a priori, the marked floor effect at 0-24 hours rescued morphine consumption, and the insufficient post hoc power (45%) limited the ability of the study to detect small but potentially clinically meaningful between-group differences. Accordingly, the absence of statistically significant differences should not be interpreted as evidence of equivalence or noninferiority, as no noninferiority margin was prespecified. The CIs presented for continuous variables describe group-specific mean estimates rather than prespecified noninferiority or equivalence margins; therefore, they should not be used to infer comparability between the 2 analgesic strategies. Fifth, although no clinical evidence of local anesthetic systemic toxicity was observed, plasma concentrations of lidocaine and bupivacaine were not measured; therefore, conclusions concerning systemic safety remain limited and should be interpreted cautiously. Future adequately powered trials with extended follow-up should validate these findings and incorporate patient-centered recovery instruments such as ObsQoR-10, as well as breastfeeding-related outcomes, maternal–infant bonding, sleep quality, and functional recovery measures.

In this exploratory, pilot randomized trial, 0-24 hours rescue morphine consumption and VAS pain scores did not significantly differ between the LWI+RAI+IPLA and ITM groups during the first 24 hours after cesarean delivery. However, the absence of statistically significant differences should not be interpreted as evidence of comparability, equivalence, or noninferiority between the 2 analgesic strategies. Intrathecal morphine provided a longer time to the first rescue opioid requirement, whereas LWI+RAI+IPLA was associated with a significantly lower incidence of pruritus, while the between-group difference in nausea/vomiting was not statistically significant. Given that the study was not designed or powered as an equivalence/noninferiority trial and that rescue morphine consumption showed a floor effect, these findings should be interpreted cautiously. Larger, adequately powered trials are warranted to confirm the comparative effectiveness and to better define the role of LWI+RAI+IPLA when neuraxial opioids are contraindicated or unavailable.

## Figures and Tables

**Figure 1.**
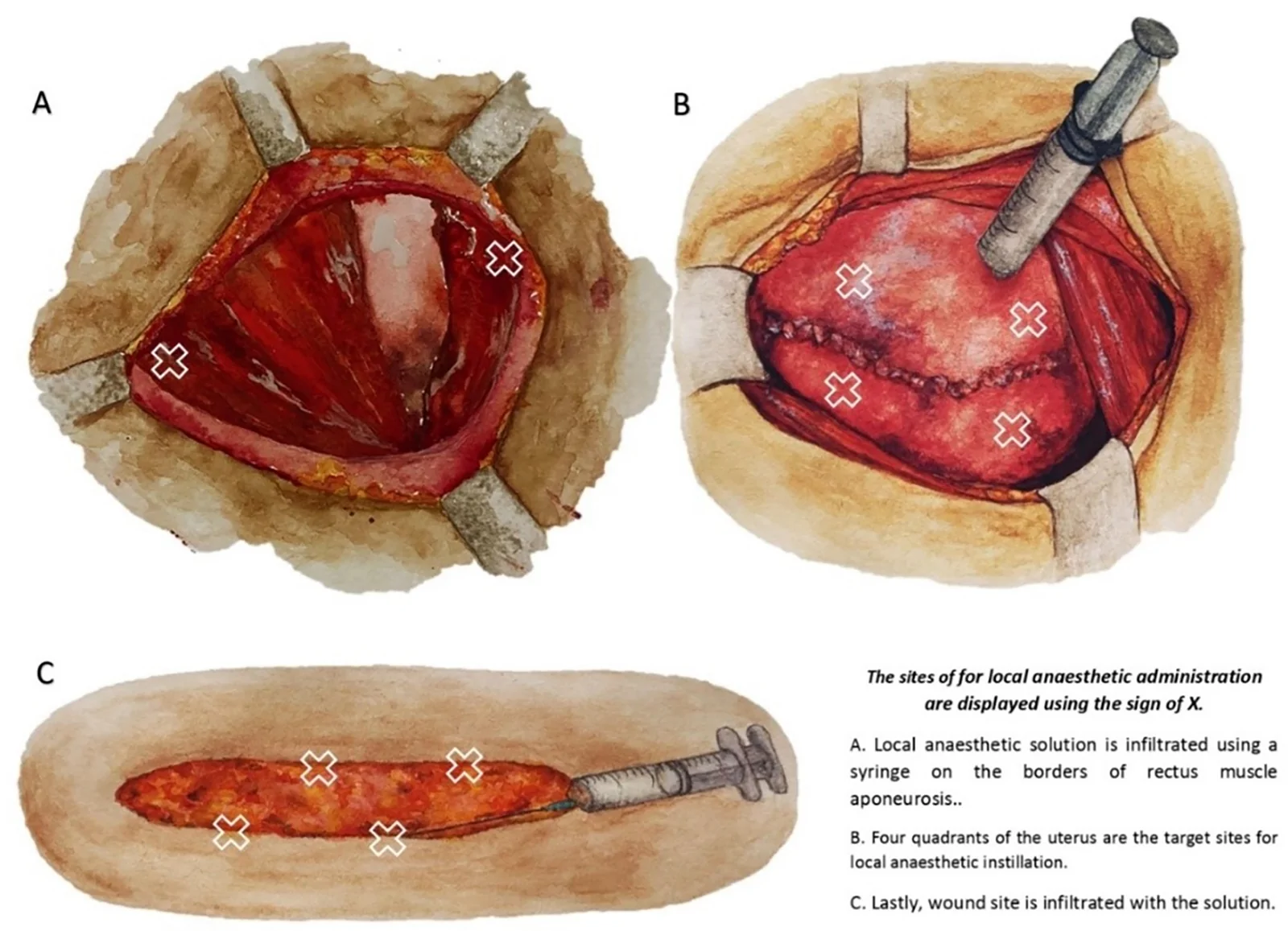
Illustration of LWI+RAI+IPLA. IPLA, intraperitoneal local anesthetic instillation; LWI, local wound infiltration; RAI, rectus aponeurosis infiltration.

**Figure 2.**
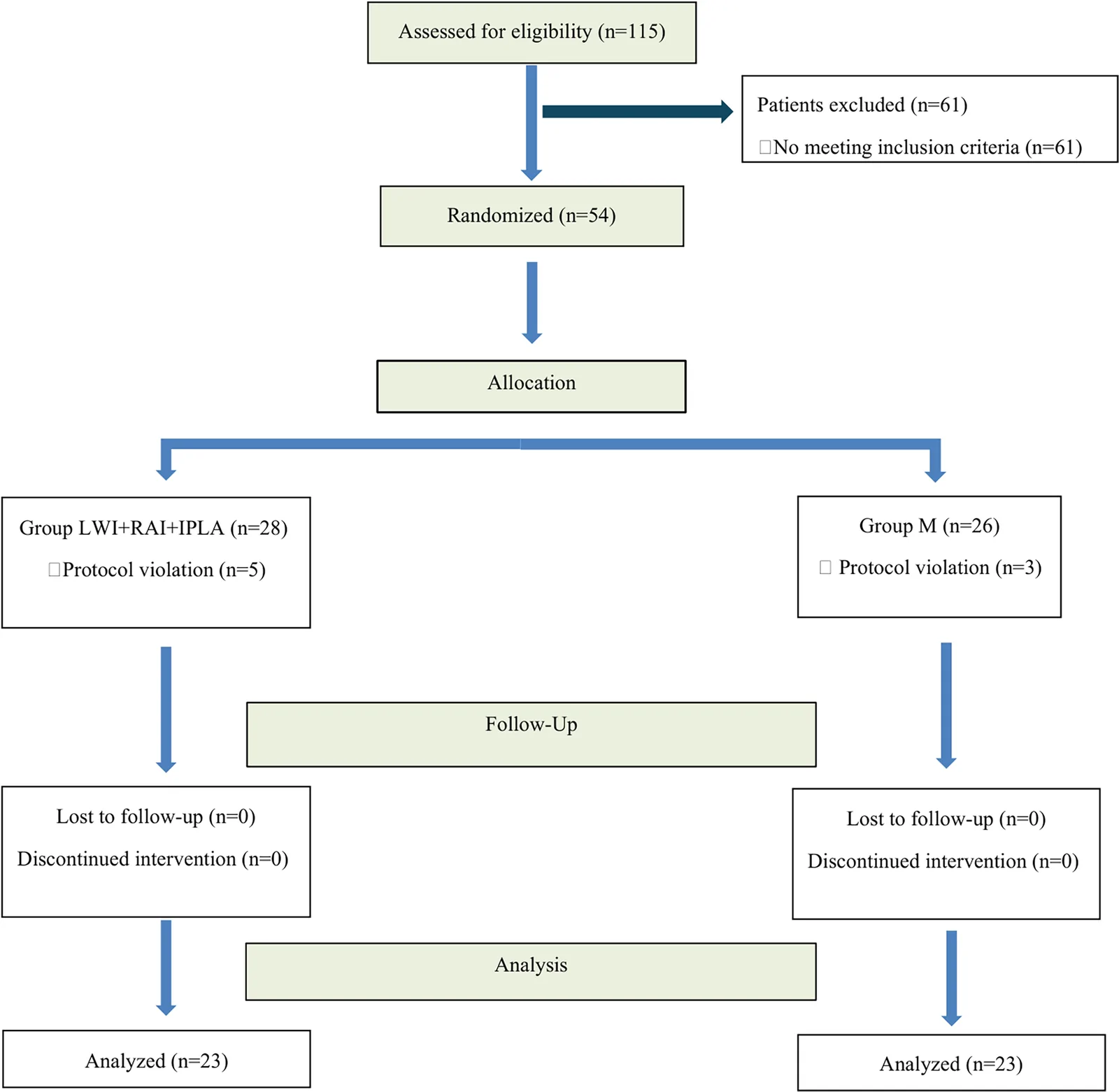
Flow chart of the study participants

**Figure 3.**
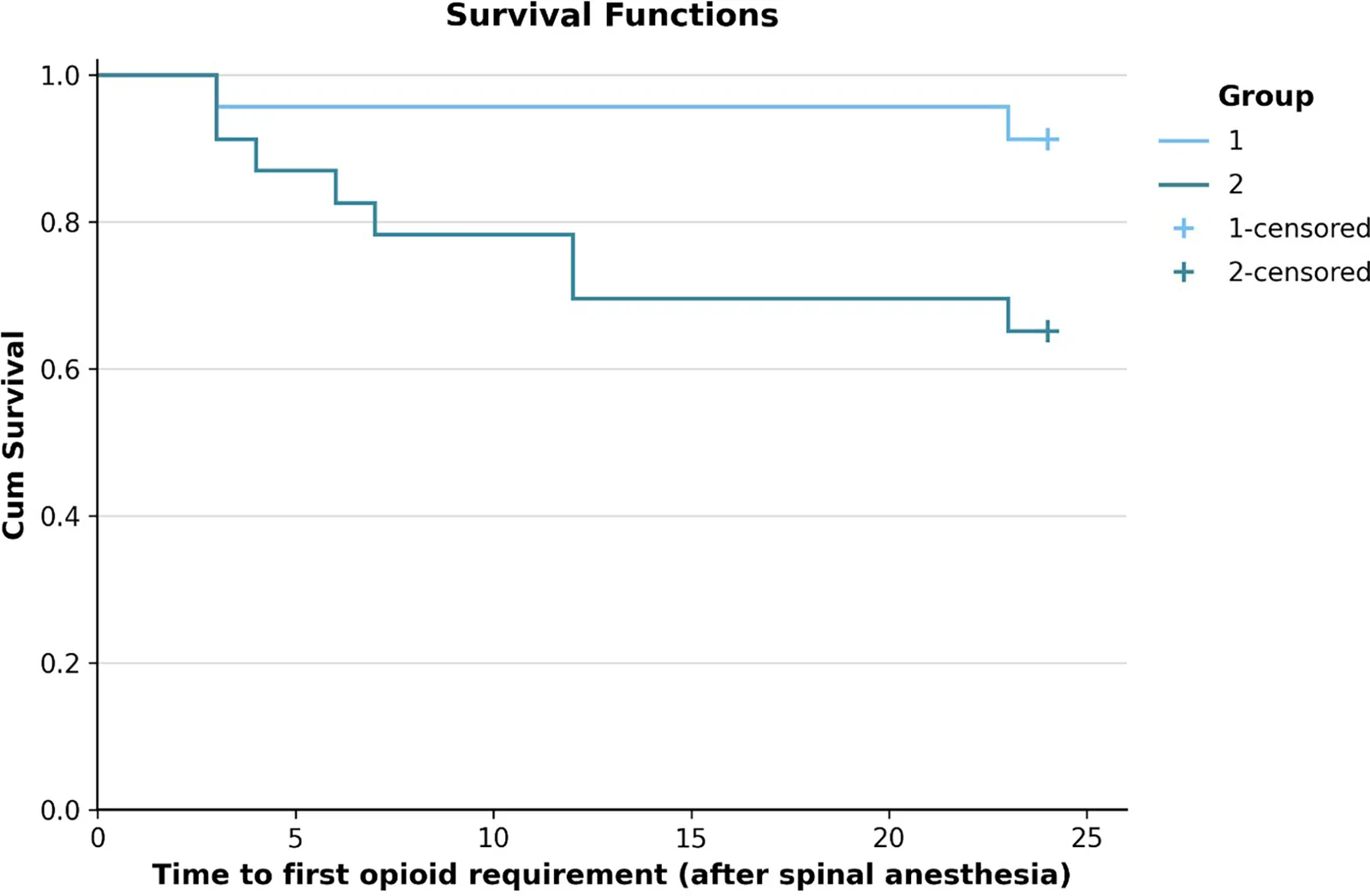
Kaplan–Meier curves for time to first postoperative rescue opioid requirement in the intrathecal morphine and LWI+RAI+IPLA groups. IPLA, intraperitoneal local anesthetic instillation; LWI, local wound infiltration; RAI, rectus aponeurosis infiltration.

**Table 1.** Patient Demographic Characteristics and Duration of Surgery

	Group M (n = 23)		Group LWI+RAI+IPLA (n = 23)		*P*	Cohen’s *d* (95% GA)	Mean Difference (95% CI)
	Mean ± SD (95% CI)	Median (min-max)	Mean ± SD (95% CI)	Median (min-max)			
Age (years)	29.65 ± 4.62 (27.65-31.65)	29 (22-40)	30.60 ± 5.36 (28.25-33.11)	29 (20-41)	.521	−0.191 (−0.769, 0.390)	−0.96 (−3.93, 2.02)
Weight (kg)	79.17 ± 8.79 (75.37-82.98)	80 (56-95)	79.73 ± 10.38 (74.72-84.00)	77 (54-95)	.843	−0.059 (−0.637, 0.520)	−0.57 (−6.28, 5.15)
Height (cm)	160.56 ± 5.55 (158.16-162.97)	160 (150-170)	164.43 ± 5.54 (161.90-166.92)	165 (155-175)	.023*	−0.697 (−1.289, −0.098)	−3.87 (−7.17, −0.57)
BMI (kg/m^2^)	30.30 ± 2.93 (29.04-31.57)	31 (24-34)	29.26 ± 3.69 (27.48-30.79)	30 (19-34)	.294	0.313 (−0.270, 0.893)	1.04 (−0.94, 3.03)
Gestational age (weeks)	37.69 ± 1.36 (37.11-38.29)	38 (34-39)	38.00 ± 0.99 (37.60-38.49)	38 (36-40)	.740	−0.255 (−0.834, 0.327)	−0.30 (−1.02, 0.41)
Duration of surgery (minutes)	45.65 ± 13.16 (39.96-51.35)	48 (21-75)	49.22 ± 13.19 (43.38-55.08)	45 (33-80)	.665	−0.271 (−0.857, 0.318)	−3.58 (−11.50, 4.35)

The values are presented as the mean ± SD (95% CI) and median (min-max). Cohen’s *d* was calculated using the pooled standard deviation.

BMI, body mass index; IPLA, intraperitoneal local anesthetic instillation; LWI, local wound infiltration, M, intrathecal morphine; RAI, rectus aponeurosis infiltration.

*P < .05.

**Table 2.** Postoperative Data and Outcomes

	Group M (n = 23)		Group LWI+RAI+IPLA (n = 23)		*P*	Cohen’s *d* (95% GA)	Mean Difference (95% CI)
	Mean ± SD (95% CI)	Median (min-max)	Mean ± SD (95% CI)	Median (min-max)			
Time to first rescue opioid requirement, restricted mean (SE), (hours)	23.04 ± 4.37 (19.20-25.66)	24 (3-24)	18.69 ± 8.35 (15.73-23.27)	24 (3-24)	.032*^k^	0.652 (0.055, 1.242)	4.348 (0.384, 8.312)
Time to first ambulation (hours)	6.66 ± 1.60 (5.46-7.25)	6 (3-9)	7.39 ± 2.06 (6.68-8.52)	7 (3-14)	.229^z^	−0.483 (−1.106, 0.146)	−1.002 (−2.323, 0.318)
Time to first bowel movement (hours)	8.26 ± 4.47 (5.98-11.16)	7 (3-22)	11.3 ± 5.86 (8.55-14.05)	9 (5-26)	.058^z^	−0.570 (−1.249, 0.118)	−3.033 (−6.733, 0.666)
Total morphine consumption (mg)	0.34 ± 0.98 (−0.24 to 1.10)	0 (0-4)	1.3 ± 2.22 (0.17-1.83)	0 (0-8)	.075^z^	−0.556 (−1.143, 0.036)	−0.957 (−1.978, 0.065)
Patient satisfaction	4.39 ± 0.65 (3.87-4.71)	4 (3-5)	4.26 ± 0.81 (3.97-4.73)	4 (3-5)	.666^z^	0.177 (−0.403, 0.755)	0.130 (−0.308, 0.569)

IPLA, intraperitoneal local anesthetic instillation; LWI, local wound infiltration, M, intrathecal morphine; RAI, rectus aponeurosis infiltration.

*P < .05. Cohen’s *d* values were calculated using the pooled standard deviation.

^z^Mann–Whitney *U-*test.

^k^Log-rank (Mantel–Cox) test. The restricted mean survival time was estimated over the 24-hour follow-up period.

**Table 3.** Postoperative VAS Scores at Rest and During Movement

	Group M (n = 23)		Group LWI+RAI+IPLA (n = 23)		*P*	Cohen’s *d* (95% GA)	Mean Difference (95% CI)
	Mean ± SD (95% CI)	Median (min-max)	Mean ± SD (95% CI)	Median (min-max)			
Rest							
2 h	0.69 ± 0.92 (0.30-1.10)	0 (0-3)	0.78 ± 1.38 (0.19-1.38)	0 (0-5)	.716^z^	−0.074 (−0.652, 0.505)	−0.087 (−0.785, 0.612)
4 h	0.78 ± 0.85 (0.41-1.15)	1 (0-2)	1.26 ± 1.60 (0.57-1.95)	1 (0-5)	.535^z^	−0.373 (−0.954, 0.212)	−0.478 (−1.240, 0.284)
6 h	0.95 ± 0.87 (0.58-1.34)	1 (0-2)	1.69 ± 1.96 (0.85-2.54)	1 (0-8)	.264^z^	−0.486 (−1.070, 0.104)	−0.739 (−1.643, 0.165)
12 h	0.91 ± 0.94 (0.50-1.32)	1 (0-2)	1.56 ± 1.27 (1.01-2.12)	2 (0-5)	.087^z^	−0.581 (−1.168, 0.013)	−0.652 (−1.320, 0.015)
24 h	1.30 ± 1.25 (0.76-1.85)	1 (0-5)	1.30 ± 1.60 (0.61-2.00)	1 (0-7)	.723^z^	0.000 (−0.578, 0.578)	0.000 (−0.858, 0.858)
Within-group *P*	.491^f^	—	.033^f^*	—	—	—	—
Post hoc	—	—	2 h-6 h	—	—	—	—
Movement							
2 h	1.21 ± 1.27 (0.66-1.77)	1 (0-5)	1.04 ± 1.69 (0.31-1.78)	0 (0-6)	.290^z^	0.116 (−0.463, 0.694)	0.174 (−0.717, 1.065)
4 h	1.34 ± 1.02 (0.90-1.79)	2 (0-3)	1.78 ± 1.80 (1.00-2.56)	1 (0-6)	.757^z^	−0.296 (−0.875, 0.287)	−0.435 (−1.309, 0.439)
6 h	1.56 ± 1.03 (1.12-2.01)	2 (0-4)	2.30 ± 2.00 (1.44-3.17)	2 (0-8)	.257^z^	−0.462 (−1.046, 0.126)	−0.739 (−1.690, 0.211)
12 h	1.52 ± 1.08 (1.05-1.99)	2 (0-3)	2.21 ± 1.53 (1.55-2.88)	2 (0-6)	.170^z^	−0.524 (−1.109, 0.067)	−0.696 (−1.485, 0.094)
24 h	2.21 ± 1.83 (1.42-3.01)	2 (0-7)	2.00 ± 1.73 (1.25-2.75)	1 (0-8)	.644^z^	0.122 (−0.457, 0.700)	0.217 (−0.842, 1.277)
Within-group *P*	.228^f^	—	.004^f^*	—	—	—	—
Post hoc	—	—	2 h-6 h; 2 h-12 h	—	—	—	—

IPLA, intraperitoneal local anesthetic instillation; LWI, local wound infiltration, M, intrathecal morphine; RAI, rectus aponeurosis infiltration; VAS, visual analog scale.

*P < .05. Cohen’s *d* values were calculated using the pooled standard deviation.

^z^Mann–Whitney *U-*test.

^f^Friedman test.

## Data Availability

The data that support the findings of this study are available on request from the corresponding author.
